# A novel frameshift variant in *TTC29* associated with total asthenozoospermia and male infertility

**DOI:** 10.3389/fcell.2026.1937470

**Published:** 2026-08-27

**Authors:** Lingyi Li, Feng Wan, Ke Feng, Xiaowei Qu, Yanqing Xia, Jianjun Dong, Yilin Liu, Yanbing Zhao, Guangli Zhu, Baona Yin, Haibin Guo

**Affiliations:** 1 Department of Reproductive Medicine Center, Henan Provincial People’s Hospital, Zhengzhou, Henan, China; 2 Department of Andrology, Jiaozuo Maternal and Child Health Hospital, Jiaozuo, Henan, China; 3 Department of Reproductive Medicine Center, Jiaozuo Maternal and Child Health Hospital, Jiaozuo, Henan, China

**Keywords:** frameshift variant, male infertility, multiple morphological abnormalities of the sperm flagella, total asthenozoospermia, TTC29

## Abstract

**Background:**

Severe sperm motility defects are an important cause of male infertility and are frequently associated with sperm flagellar abnormalities. *TTC29* encodes an evolutionarily conserved axonemal protein implicated in sperm flagellar assembly and motility, yet the pathogenic mechanisms of *TTC29* variants remain incompletely understood.

**Methods:**

A Chinese man with total asthenozoospermia was investigated using whole-exome sequencing, Sanger sequencing, transmission electron microscopy, sperm immunofluorescence, and heterologous expression analysis in HEK293 T cells. The clinical outcome following intracytoplasmic sperm injection (ICSI) was also evaluated.

**Results:**

Whole-exome sequencing identified a novel *TTC29* frameshift variant (NM_031956.4:c.121dupA; p. Ile41Asnfs*21), which was confirmed by Sanger sequencing. Transmission electron microscopy revealed severe ultrastructural abnormalities involving both the midpiece and principal piece, with disruption of the canonical 9 + 2 axonemal architecture and marked abnormalities of peri-axonemal structures. In representative immunofluorescence images, TTC29 signal was readily detected along the sperm flagellum in the fertile control, whereas a visibly weaker signal was observed in spermatozoa from the proband. Heterologous expression analysis showed little or no detectable production of the expected TTC29 protein product from the mutant construct. Although fertilization was achieved following two ICSI cycles, no clinical pregnancy was obtained.

**Conclusion:**

We identified a novel *TTC29* frameshift variant associated with total asthenozoospermia and male infertility. Genetic, ultrastructural, and heterologous expression evidence supports a deleterious effect of the identified *TTC29* variant, thereby expanding the mutational spectrum of *TTC29*-related infertility and providing further insight into the molecular basis of severe sperm motility disorders.

## 1 Introduction

Infertility is a major reproductive health problem worldwide and affects approximately 10%–15% of couples of reproductive age. Male factors account for, or contribute to, nearly half of infertility cases (Krausz and Riera-Escamilla, 2018; Carson and Kallen, 2021). Among the diverse causes of male infertility, severe impairment of sperm motility is clinically important because it compromises the ability of spermatozoa to migrate through the female reproductive tract, interact with the oocyte, and complete fertilization (Coutton et al., 2015; Krausz and Riera-Escamilla, 2018).

Multiple morphological abnormalities of the sperm flagella (MMAF) represent a severe form of asthenoteratozoospermia. This phenotype is characterized by heterogeneous flagellar defects, including absent, short, bent, coiled, and irregular-caliber flagella, and is usually accompanied by markedly reduced or completely absent sperm motility (Coutton et al., 2015; Sironen et al., 2020; Wang et al., 2020). Most men with MMAF present with primary infertility and have a very low probability of natural conception; therefore, intracytoplasmic sperm injection (ICSI) is commonly required to achieve fertilization (Palermo, 1992; Wambergue et al., 2016).

The sperm flagellum is a highly organized organelle. Its core structure is the conserved 9 + 2 axoneme, which consists of nine peripheral microtubule doublets surrounding a central pair of microtubules. Coordinated movement also depends on dynein arms, radial spokes, outer dense fibers, and the fibrous sheath (Satir and Christensen, 2007; Inaba, 2011). Beyond this structural scaffold, intraflagellar transport and the coordinated assembly of multiple protein complexes are required for late spermiogenesis, flagellar elongation, and maintenance of flagellar function (Taschner and Lorentzen, 2016; Lehti and Sironen, 2017). Defects in any of these processes can result in flagellar dysplasia and severe sperm motility failure.

MMAF is genetically heterogeneous. With the increasing use of whole-exome sequencing (WES), pathogenic variants have been identified in genes encoding dynein heavy chains, axonemal structural proteins, fibrous sheath components, and proteins involved in intraflagellar transport or flagellar assembly. These genes include *DNAH1*, *DNAH2*, *CFAP43*, *CFAP44*, *CFAP69*, *CFAP65*, *FSIP2*, *AK7*, *WDR66*, *SPEF2*, *TTC21A*, and *TTC29* (Ben Khelifa et al., 2014; Amiri-Yekta et al., 2016; Tang et al., 2017; Dong et al., 2018; Liu C. et al., 2019; Lorès et al., 2019; Nsota Mbango et al., 2019; Li et al., 2020; Dai et al., 2022; Dacheux et al., 2023; Zhou et al., 2024). Nevertheless, the molecular diagnosis remains elusive in a subset of patients with severe sperm flagellar defects, indicating that additional disease-causing variants and genotype-phenotype relationships remain to be defined.

*TTC29* encodes tetratricopeptide repeat domain 29, a protein containing tetratricopeptide repeat (TPR) motifs. TPR motifs commonly mediate protein-protein interactions and participate in multiprotein complex assembly (Blatch and Lässle, 1999). Previous studies have shown that biallelic *TTC29* variants cause severe asthenozoospermia, MMAF, and male infertility. TTC29 localizes to the sperm flagellum and is thought to participate in axonemal integrity and intraflagellar transport-related processes (Liu C. et al., 2019; Lorès et al., 2019; Dai et al., 2022). In addition, ZMYND12 has been reported to interact with TTC29 and DNAH1 within an axoneme-associated protein network, further suggesting a role for TTC29 in maintaining sperm flagellar function (Dacheux et al., 2023).

In this study, we identified a novel *TTC29* frameshift variant, c.121dupA (p.Ile41Asnfs*21), in an infertile man with total asthenozoospermia. We evaluated the available familial segregation evidence, sperm flagellar ultrastructure, TTC29 localization in spermatozoa, and the effect of c.121dupA on production of the expected TTC29 expression product in a heterologous cell system. The findings provide additional evidence for the involvement of *TTC29* loss of function in severe sperm motility defects and expand the variant spectrum of *TTC29*-associated male infertility.

## 2 Materials and methods

### 2.1 Ethics approval and informed consent

This study was approved by the Ethics Committee of Henan Provincial People’s Hospital (approval number: SYSZ-LL-2019110401). Written informed consent was obtained from all participants. The study was conducted in accordance with the Declaration of Helsinki, and all clinical data and biological samples were anonymized to protect participant privacy.

### 2.2 Study subject

A Chinese man who presented with primary infertility after 4 years of unprotected intercourse was enrolled. Based on semen analysis and clinical evaluation at Henan Provincial People’s Hospital, the proband was diagnosed with total asthenozoospermia. Bilateral testicular volume, sex hormone levels, karyotype analysis, and Y-chromosome microdeletion screening were unremarkable. The proband reported no chronic sinusitis, recurrent respiratory infection, bronchiectasis, situs inversus, or other clinical features suggestive of primary ciliary dyskinesia. His father had no known reproductive disorder. His mother had died in an accident during his childhood and was unavailable for genetic testing. The proband was an only child, and no informative maternal relatives were available for additional segregation analysis.

### 2.3 Semen analysis and sperm morphology

Semen was collected by masturbation after 3–7 days of sexual abstinence. After liquefaction, routine semen analysis was performed according to the fifth edition of the WHO laboratory manual for the examination and processing of human semen (WHO, 2010), which was the validated laboratory standard in use at our reproductive medicine center at the time of the patient’s clinical evaluation in 2023. Semen volume, pH, sperm concentration, total sperm count, progressive motility, non-progressive motility, and the proportion of immotile spermatozoa were recorded. Sperm morphology was examined after staining, with emphasis on flagellar defects such as absent, short, bent, coiled, and irregular-caliber flagella.

### 2.4 Whole-exome sequencing and variant prioritization

Peripheral blood samples (5 mL each) were collected from the proband and his father. Genomic DNA was extracted using the DNeasy Blood & Tissue Kit (Qiagen, Hilden, Germany). DNA concentration and purity were assessed using a Qubit 4.0 fluorometer (Thermo Fisher Scientific, Waltham, MA, United States of America) and a NanoDrop One spectrophotometer (Thermo Fisher Scientific). DNA samples with a concentration >50 ng/μL and an A260/A280 ratio of 1.8–2.0 were used for sequencing. WES was performed using the IDT xGen Exome Research Panel V1.0 (Integrated DNA Technologies, Coralville, IA, United States of America) for target enrichment, followed by paired-end 150-bp sequencing on an Illumina NovaSeq 6,000 platform. Raw reads were assessed using FastQC v0.11.9, with Q30 > 90% and an average sequencing depth ≥100× as quality-control criteria. Quality-filtered reads were aligned to the human reference genome GRCh37/hg19 using BWA-MEM v0.7.17. GATK v4.2.6.1 was used for duplicate marking, base quality score recalibration, and variant calling. Variant annotation was performed with ANNOVAR (2019Oct24), integrating population frequency and clinical annotation data from gnomAD v2.1.1, dbSNP v153, 1,000 Genomes, ExAC, and ClinVar. Synonymous variants without predicted effects on splicing were excluded. Candidate variants were prioritized if they were located in coding regions or canonical splice-site regions, had a minor allele frequency (MAF) < 0.001 in population databases, were predicted to affect protein structure or function, were located in genes associated with severe sperm motility defects or flagellar abnormalities, and were compatible with or suggestive of autosomal recessive inheritance. For the identified *TTC29* variant, population frequency was additionally reassessed in gnomAD v2.1.1 using both the global population and the East Asian subpopulation. Variant pathogenicity was interpreted according to the American College of Medical Genetics and Genomics/Association for Molecular Pathology guidelines (Richards et al., 2015). To evaluate the possibility of pseudohomozygosity, the original WES data were retrospectively reanalyzed using an exon-level read-depth-based copy-number method. Normalized sequencing coverage was assessed across the coding exons of *TTC29*, with particular attention to exon 4 and the adjacent exons.

### 2.5 Sanger sequencing validation

Specific primers were designed to amplify the genomic region containing the candidate *TTC29* variant c.121dupA. The primers were based on the *TTC29* exon 4 reference sequence in GRCh37/hg19: forward primer 5′-ATG​CTG​GAC​CTG​GTG​TTC​CT-3′ and reverse primer 5′-GCC​AGT​GAC​AGC​CAC​ATT​GA-3′. Primer specificity was evaluated using NCBI Primer-BLAST. Purified PCR products were subjected to Sanger sequencing, and the sequencing traces were compared with the *TTC29* reference sequence using SnapGene v4.3 to assess segregation of the c.121dupA variant in the family.

### 2.6 Transmission electron microscopy

Semen samples were obtained from the proband and a fertile control. The fertile control had semen parameters within the normal reference ranges and a history of natural conception. After liquefaction, sperm pellets were collected by centrifugation and fixed in 2.5% glutaraldehyde in phosphate buffer (pH 7.4) at 4 °C for 4 h. The proband and control samples were processed using the same fixation, dehydration, embedding, sectioning, and staining procedures. After washing in phosphate buffer, the samples were post-fixed with 1% osmium tetroxide. The specimens were then dehydrated through a graded ethanol series, gradually infiltrated with acetone–epoxy resin mixtures, embedded in pure epoxy resin, and polymerized according to standard procedures. Ultrathin sections of approximately 70 nm were prepared using an ultramicrotome and stained with uranyl acetate and lead citrate. Images were acquired using a JEM-1400 transmission electron microscope (JEOL, Japan) at an accelerating voltage of 80 kV.

Cross-sections of the sperm flagella were evaluated with particular attention to the ultrastructure of the midpiece and principal piece. The assessed structures included the canonical “9 + 2” axonemal organization, central pair of microtubules (CP), peripheral doublet microtubules (DMTs), outer dense fibers (ODFs), mitochondrial sheath (MS), and fibrous sheath (FS), with emphasis on their integrity and spatial organization. For quantitative ultrastructural analysis, all available non-overlapping and clearly interpretable transverse sections of the sperm flagellum were systematically evaluated. Cross-sections were classified as midpiece or principal-piece sections according to their characteristic peri-axonemal structures, including the mitochondrial sheath in the midpiece and the fibrous sheath in the principal piece. Because the proband’s spermatozoa predominantly exhibited absent, short, and coiled flagella, clearly identifiable end-piece cross-sections were rarely encountered and were insufficient for a meaningful quantitative comparison; therefore, end-piece sections were excluded from the analysis. Each cross-section was assigned to one of four mutually exclusive categories: isolated central-pair (CP) abnormality, isolated peripheral doublet microtubule (DMT) abnormality, complex axonemal and/or peri-axonemal disorganization, or preserved ultrastructural architecture. Isolated CP abnormality was defined as absence or poor definition of the CP with the peripheral DMTs remaining largely preserved. Isolated DMT abnormality was defined as loss or marked disorganization of one or more peripheral DMTs with the CP remaining identifiable and without concurrent multicomponent structural defects. Cross-sections showing concurrent abnormalities of the CP and DMTs, or defects involving multiple axonemal and/or peri-axonemal components, including the outer dense fibers, mitochondrial sheath, or fibrous sheath, were classified as complex disorganization. Oblique, longitudinal, poorly preserved, duplicated, or unclassifiable sections were excluded. Results were expressed as n/N (%).

### 2.7 Plasmid construction and validation

Total RNA was extracted from peripheral blood of a healthy donor using the TIANGEN RNAsimple Kit, and cDNA was synthesized using the TransGen First-Strand Synthesis Kit. The full-length coding sequence of *TTC29* was amplified using high-fidelity DNA polymerase (TaKaRa PrimeSTAR). The PCR product was digested with restriction enzymes and ligated into the pEGFP-N1 vector to generate a wild-type *TTC29* expression construct. The ligation product was transformed into *Escherichia coli* DH5α competent cells, and positive clones were verified by Sanger sequencing. A mutant construct carrying c.121dupA was generated using the Vazyme Mut Express II Fast Mutagenesis Kit V2. After DpnI digestion to remove template plasmids, the PCR product was transformed into DH5α competent cells. The presence of the desired variant and the reading frame were confirmed by Sanger sequencing. Both expression constructs were generated from intron-free cDNA and encoded a C-terminal EGFP tag. Therefore, this expression system was designed to assess production of the expected TTC29 expression product rather than endogenous transcript processing or nonsense-mediated mRNA decay.

### 2.8 Cell culture and transfection

HEK293 T cells were recovered from liquid nitrogen and cultured in Dulbecco’s modified Eagle’s medium supplemented with 10% fetal bovine serum at 37 °C in a humidified incubator with 5% CO2. When cells reached 80%–90% confluence, they were seeded into six-well plates and transfected with empty vector, wild-type TTC29-EGFP, or mutant TTC29-EGFP constructs using Lipofectamine 2000 (Life Technologies). Lipofectamine 2000 and plasmids were diluted in Opti-MEM (Gibco), mixed, incubated at room temperature for 20 min, and added dropwise to the culture medium. EGFP fluorescence was observed 48 h after transfection, and cells were collected for Western blotting.

### 2.9 Western blotting

At 48 h after transfection, cells were washed twice with cold PBS and lysed on ice for 30 min in RIPA lysis buffer (P0013B; Beyotime Biotechnology) supplemented with protease inhibitors. Equal amounts of protein were mixed with 6× SDS-PAGE loading buffer, denatured by boiling, separated on 10% SDS-PAGE gels, and transferred to PVDF membranes. Membranes were blocked with 5% non-fat milk for 1 h at room temperature and washed three times with TBST. The membranes were incubated overnight at 4 °C with anti-GFP antibody (300,943; Zenbio; 1:2000) or anti-TTC29 antibody (R86014; Sigma-Aldrich; 1:2000). After washing, membranes were incubated with horseradish peroxidase-conjugated secondary antibody (1:1,000) for 1 h at room temperature. Protein bands were detected using ECL chemiluminescence reagent (P0018; Beyotime Biotechnology) and imaged using a ChemiDoc XRS system (Bio-Rad). GAPDH served as the loading control.

### 2.10 Sperm immunofluorescence

Sperm pellets were collected by centrifugation and fixed with 4% paraformaldehyde at 4 °C for 30 min. After three washes with PBS, spermatozoa were resuspended, spread onto 14-mm poly-D-lysine-coated coverslips (LABSELECT), and air dried. Samples were permeabilized with 0.3% Triton X-100 (Sigma-Aldrich) for 20 min at room temperature and blocked with 5% bovine serum albumin (Gibco) for 1 h. The samples were incubated overnight at 4 °C with rabbit polyclonal anti-TTC29 antibody (R86014; Sigma-Aldrich; 1:100) and anti-α-tubulin antibody (T7451; Sigma-Aldrich; 1:500). After washing, samples were incubated with Alexa Fluor 488- and Alexa Fluor 594-conjugated secondary antibodies (Thermo Fisher Scientific; 1:1,000) for 2 h in the dark at room temperature. Nuclei were stained with DAPI. Images were captured using a Zeiss LSM 710 confocal microscope.

### 2.11 ICSI treatment and outcome assessment

The couple underwent controlled ovarian stimulation. Follicular development was monitored by transvaginal ultrasonography, and the gonadotropin dose was adjusted according to follicular response and serum hormone levels. When trigger criteria were met, recombinant human chorionic gonadotropin was administered. Oocyte retrieval was performed 32–36 h later under transvaginal ultrasound guidance. On the day of oocyte retrieval, semen was collected by masturbation and processed in the laboratory. Viable spermatozoa were selected using the hypo-osmotic swelling test (HOST) and used for ICSI. Embryos were cultured under standard conditions and evaluated daily. Day-3 embryos were assessed based on blastomere number, blastomere symmetry, degree of cytoplasmic fragmentation, and other relevant morphological features. Embryo transfer was performed on day 3 or day 5 according to embryo development and quality. Serum β-hCG was measured 2 weeks after transfer, and clinical pregnancy was confirmed by transvaginal ultrasound approximately 4 weeks after transfer.

## 3 Results

### 3.1 Clinical characteristics of the proband

The proband was a 36-year-old man who presented with primary infertility after 4 years of unprotected intercourse. Semen analysis showed a semen volume of 1.5 mL, pH 7.5, sperm concentration of 18.6 × 10^6/mL, progressive motility of 0%, non-progressive motility of 0%, and 100% immotile spermatozoa. The total sperm count was 27.9 × 10^6 per ejaculate. Sperm viability was 44%, below the lower reference value of 58%, indicating that a proportion of the completely immotile spermatozoa remained viable (Table 1). Bilateral testicular volume was normal, and seminal plasma biochemistry, sex hormone levels, karyotype analysis (46,XY), and Y-chromosome AZF microdeletion screening were unremarkable. The proband had no history of chronic respiratory infection, situs inversus, sinusitis, or other typical features of primary ciliary dyskinesia. Sperm morphology analysis showed marked flagellar abnormalities, including short, bent, coiled, and absent flagella, consistent with severe sperm flagellar defects.

**TABLE 1:** Clinical and semen characteristics of the patient.

Subjects	Patient	References range
Age (years)	36	-
Semen volume (mL)	1.5	≥1.5
Semen pH	7.5	≥7.2
Semen concentration (10^6^/mL)	18.6	≥15
Total sperm count (10^6^/ejaculate)	27.9	≥39
Progressive motility (%)	0	≥32
Sperm viability (%)	44	≥58
Normal spermatozoa (%)	1.0	≥4
Defective spermatozoa (%)	99.0	-

Reference values for semen parameters are based on the fifth edition of the WHO (2010).

### 3.2 Identification and familial validation of the *TTC29* variant

**FIGURE 1:**
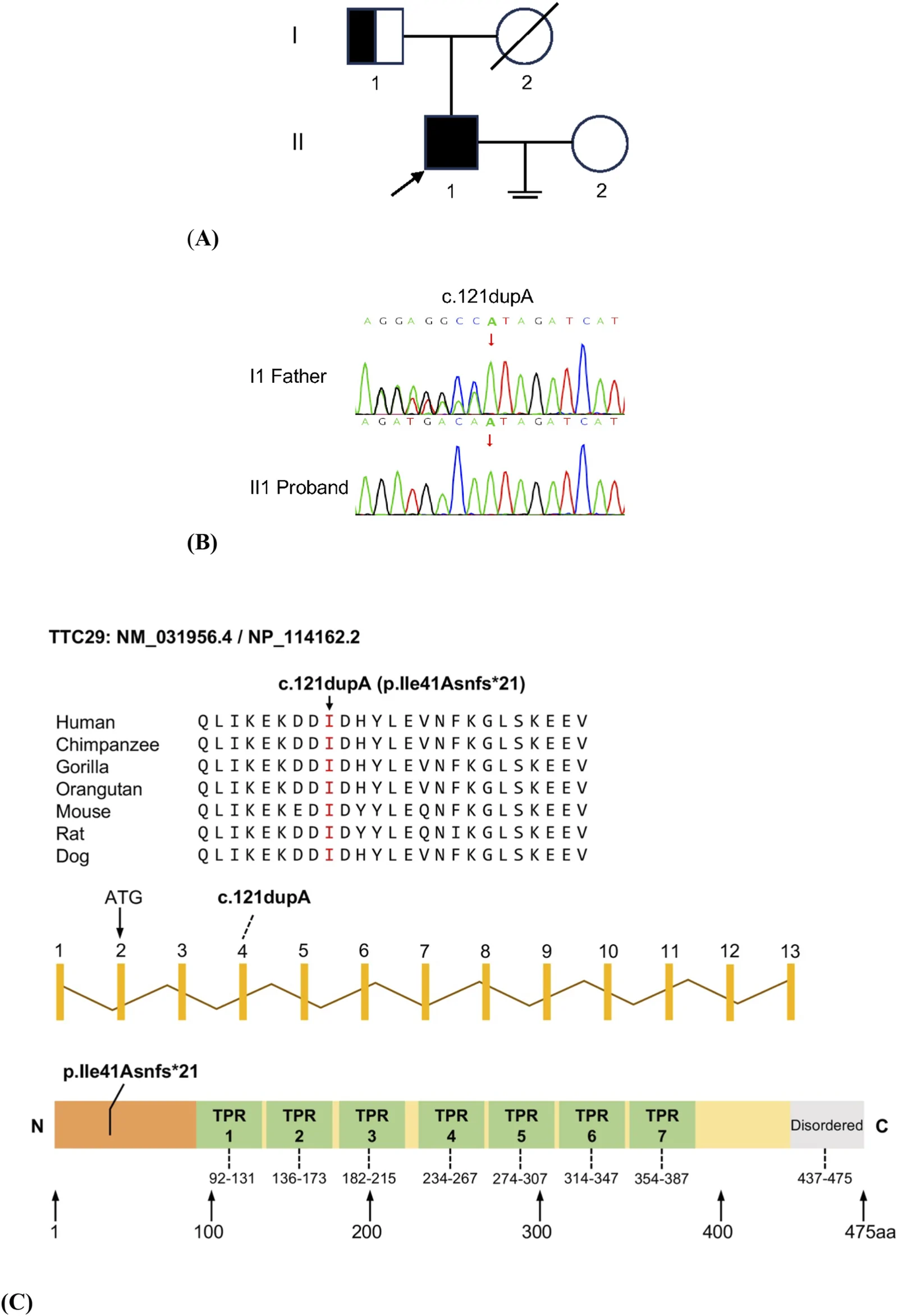
Identification of the *TTC29* c.121dupA variant **(A)** Pedigree of the family. The proband is indicated by an arrow **(B)** Sanger sequencing confirmed the *TTC29* c.121dupA variant in an apparently homozygous state in the proband and in a heterozygous state in his father **(C)** Multiple-sequence alignment showing conservation of the affected residue among species, together with a schematic representation of the *TTC29* gene and protein structure showing that **(C)** 121dupA is located in exon 4 and upstream of the predicted TPR domains.

WES identified the *TTC29* frameshift variant (NM_031956.4: c.121dupA) in an apparently homozygous state in the proband. The variant is located in exon 4 and is predicted to change isoleucine at position 41 to asparagine, followed by a frameshift and premature termination (p.Ile41Asnfs*21). Sanger sequencing showed an apparently homozygous sequence pattern for c.121dupA in the proband, whereas his father carried the variant in the heterozygous state (Figures 1A,B). The paternal carrier status was compatible with autosomal recessive inheritance; however, complete trio segregation analysis could not be performed because the mother had died and no maternal biological sample was available. To evaluate the possibility that the apparently homozygous genotype resulted from a deletion involving the second allele, the original WES data were retrospectively reanalyzed for exon-level copy-number abnormalities. No reduction in normalized coverage or copy-number abnormality was identified across the coding exons of *TTC29*, including exon 4 and the adjacent exons. These findings showed no evidence of a deletion involving *TTC29* and argued against pseudohomozygosity caused by a large deletion on the second allele. The variant lies near the N-terminus of the coding sequence and is predicted to introduce a premature termination codon upstream of all predicted TPR domains. The c.121dupA variant was not detected in gnomAD v2.1.1, either in the global population or in the East Asian subpopulation. According to the ACMG/AMP guidelines and ClinGen Sequence Variant Interpretation recommendations, the variant was classified as likely pathogenic based on PVS1 and PM2_Supporting. The predicted loss-of-function effect of the early frameshift variant supported PVS1, whereas its absence from population databases supported PM2_Supporting. The phenotypic and functional findings described below provided additional support for a deleterious effect of the variant. Multiple-sequence alignment showed that the amino acid residue affected by p. Ile41Asnfs*21 is conserved among different mammalian species. Schematic analysis of the *TTC29* gene and protein structure indicated that c.121dupA is located in exon 4 and affects an N-terminal region upstream of the TPR domains, supporting a loss-of-function effect of the variant (Figure 1C).

### 3.3 Sperm morphology and transmission electron microscopy

**FIGURE 2:**
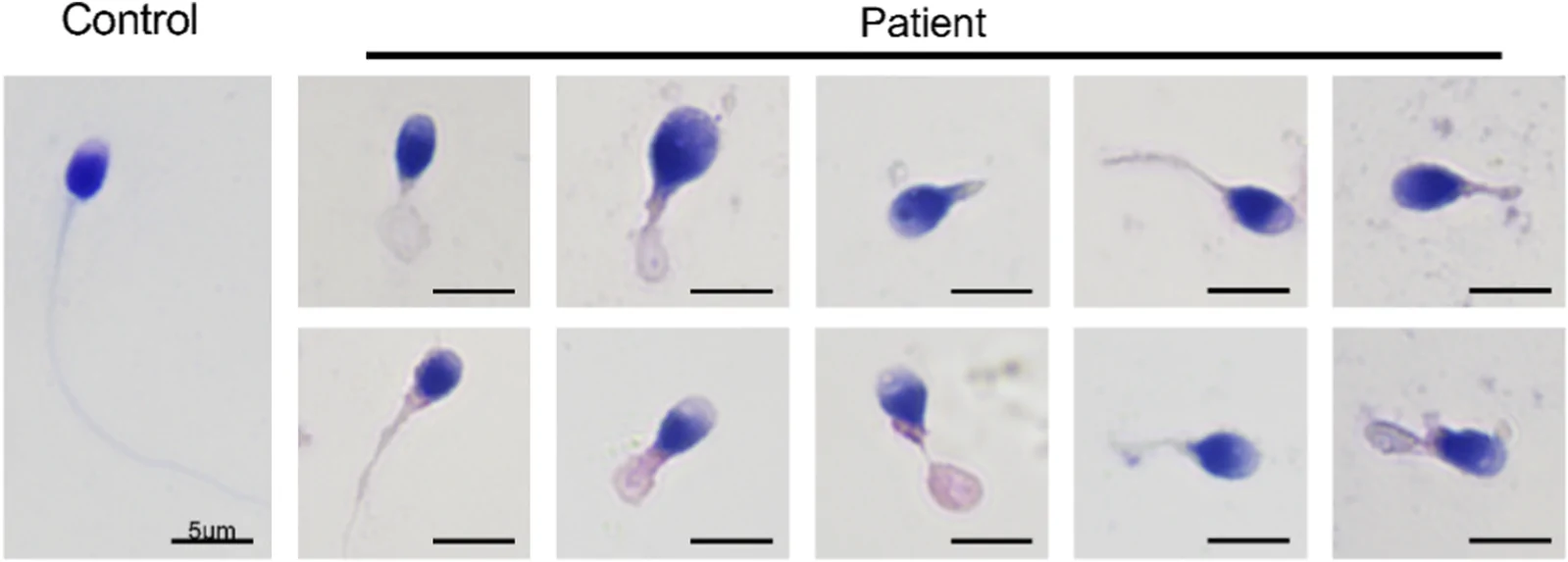
Sperm morphology of the control and the patient. Light microscopy showed normal sperm flagellar morphology in the fertile control and multiple flagellar abnormalities in the patient, including short, bent, coiled, and absent flagella.

Light microscopy showed severe sperm flagellar abnormalities in the proband, including short, bent, coiled, and absent flagella (Figure 2). To further evaluate the effect of the *TTC29* c.121dupA variant on sperm flagellar ultrastructure, transmission electron microscopy was performed on cross-sections of sperm flagella from a fertile control and the proband.

**FIGURE 3:**
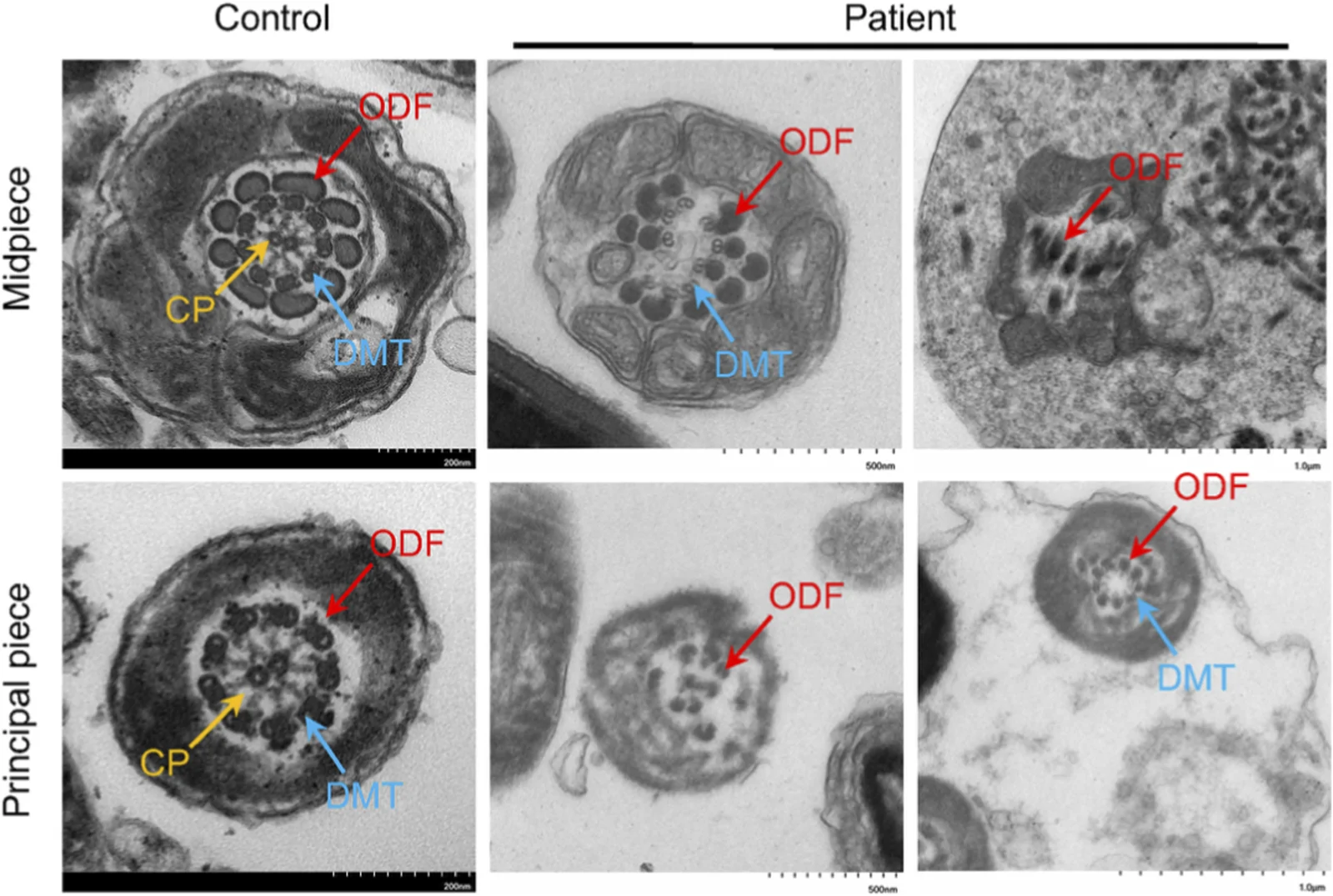
Transmission electron microscopy analysis of sperm flagellar ultrastructure. Representative transverse sections of the midpiece and principal piece from a fertile control and the proband are shown. Control sperm flagella exhibited the canonical “9 + 2” axonemal organization, whereas sperm flagella from the proband showed loss or poor definition of the central pair, loss or disorganization of peripheral doublet microtubules, and abnormal arrangement of outer dense fibers. Yellow arrows indicate the central pair (CP), blue arrows indicate peripheral doublet microtubules (DMTs), and red arrows indicate outer dense fibers (ODFs).

In the control spermatozoa, midpiece cross-sections showed the canonical “9 + 2” axonemal organization, with nine peripheral doublet microtubules surrounding a central pair of microtubules. The outer dense fibers were regularly arranged around the axoneme, and the mitochondrial sheath was continuous and well organized. Control principal-piece cross-sections also displayed a preserved “9 + 2” axoneme, regularly arranged peripheral doublet microtubules and outer dense fibers, and an intact, relatively symmetrical fibrous sheath. In contrast, the proband’s sperm flagella exhibited marked ultrastructural abnormalities in both the midpiece and principal piece. Midpiece cross-sections showed an irregular mitochondrial sheath, eccentric axonemes, disorganized peripheral doublet microtubules, and misarranged outer dense fibers. The central pair was absent or poorly defined, and the canonical “9 + 2” axonemal architecture was disrupted. In principal-piece cross-sections, the fibrous sheath was asymmetric, locally thickened, deformed, or disorganized. The axoneme appeared eccentric, collapsed, or poorly defined, with abnormal spatial arrangement of peripheral doublet microtubules and outer dense fibers. The central pair was also absent or difficult to identify (Figure 3).

Quantitative ultrastructural analysis included 75 midpiece and 75 principal-piece cross-sections from the fertile control and 77 midpiece and 69 principal-piece cross-sections from the proband. Clearly identifiable end-piece cross-sections were rarely encountered in the proband owing to the predominance of absent, short, and coiled flagella and were therefore not included in the quantitative analysis. Cross-section quantification showed that 96.1% (74/77) of the proband’s midpiece sections and 97.1% (67/69) of the principal-piece sections were abnormal, compared with 5.3% (4/75) and 4.0% (3/75), respectively, in the fertile control. In the proband’s midpiece, isolated CP abnormalities accounted for 40.3% (31/77) of the sections, isolated DMT abnormalities for 20.8% (16/77), and complex axonemal and peri-axonemal disorganization for 35.1% (27/77); only 3.9% (3/77) retained preserved ultrastructural architecture. In the principal piece, isolated CP abnormalities accounted for 49.3% (34/69), isolated DMT abnormalities for 18.8% (13/69), and complex disorganization for 29.0% (20/69); only 2.9% (2/69) retained preserved architecture. Complex abnormalities involved variable combinations of defects affecting the CP, DMTs, ODFs, mitochondrial sheath in the midpiece, and/or fibrous sheath in the principal piece.

Together, the qualitative and quantitative TEM findings demonstrated extensive abnormalities of both the axonemal core and peri-axonemal structures in the proband’s sperm flagella, involving the mitochondrial sheath in the midpiece and the fibrous sheath in the principal piece.

### 3.4 Sperm immunofluorescence analysis

**FIGURE 4:**
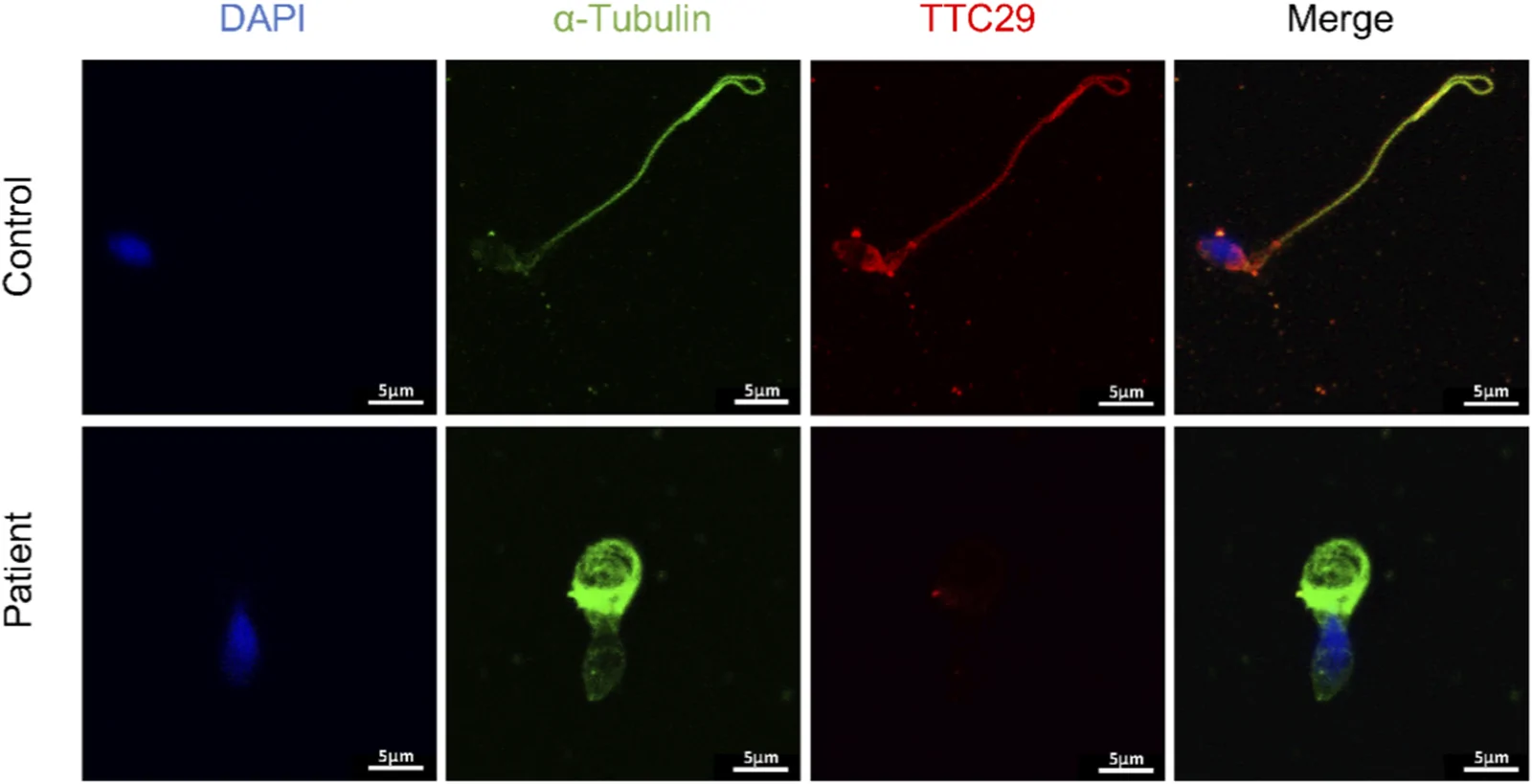
Representative immunofluorescence images of TTC29 in spermatozoa from the fertile control and the proband. TTC29 signal was readily detected along the sperm flagellum in the fertile control, whereas a visibly weaker signal was observed in representative spermatozoa from the proband. α-Tubulin labeled the sperm flagellum, and DAPI stained the nuclei. The images are presented as qualitative observations and were not used for fluorescence-intensity quantification.

In representative immunofluorescence images, TTC29 signal was readily detected along the sperm flagellum in the fertile control and partially overlapped with the α-tubulin signal. In contrast, a visibly weaker TTC29 signal was observed in representative spermatozoa from the proband, whereas α-tubulin continued to label the residual abnormal flagellar structures (Figure 4). These representative images qualitatively suggested reduced TTC29 localization or abundance in the proband’s spermatozoa.

### 3.5 Heterologous expression analysis

**FIGURE 5:**
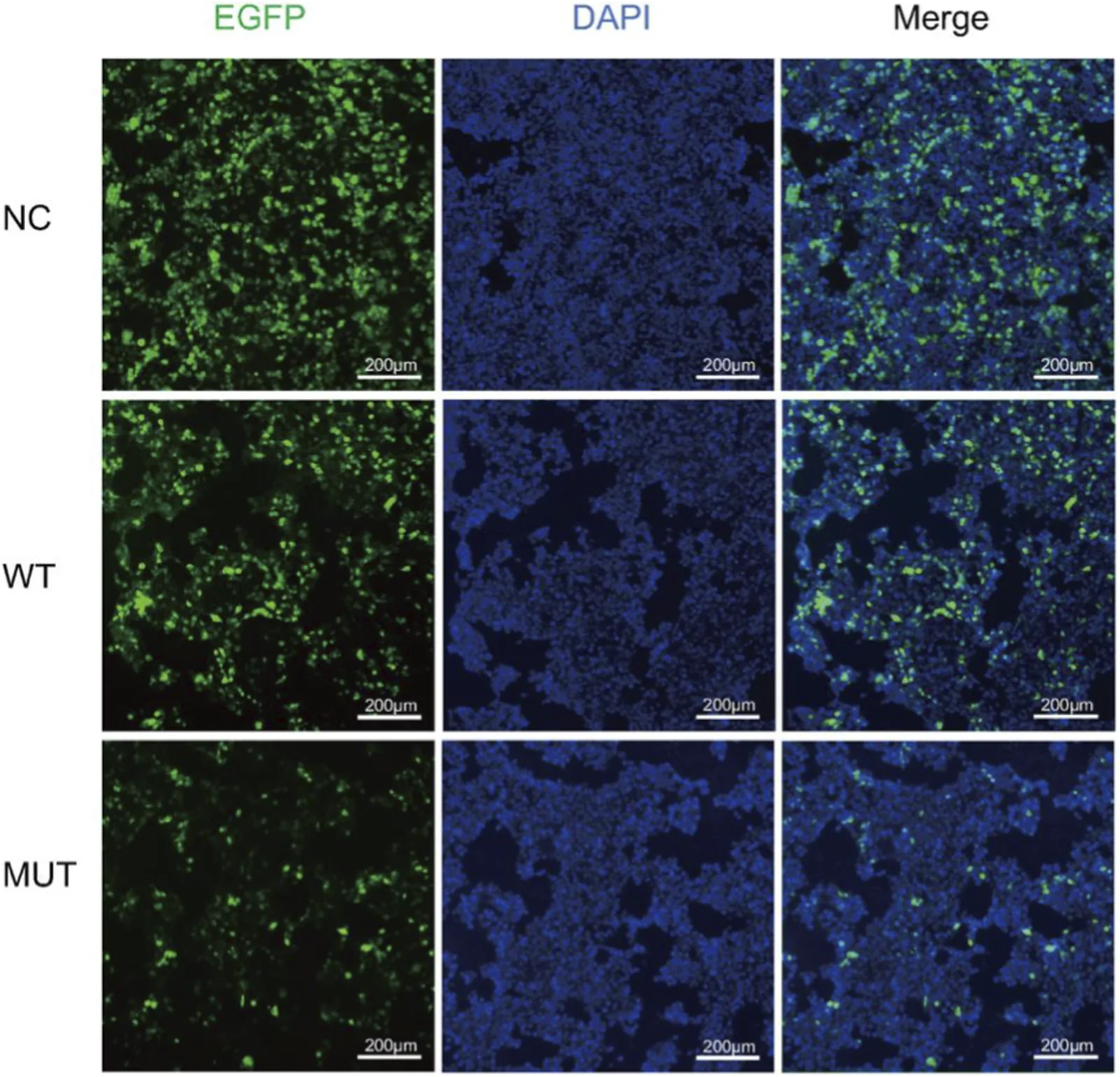
Fluorescence analysis of TTC29-EGFP expression in HEK293 T cells. HEK293 T cells were transfected with empty vector (NC), wild-type TTC29-EGFP (WT), or mutant TTC29-EGFP carrying c.121dupA (MUT). EGFP fluorescence was markedly weaker in the MUT group than in the WT group. DAPI stained the nuclei.

**FIGURE 6:**
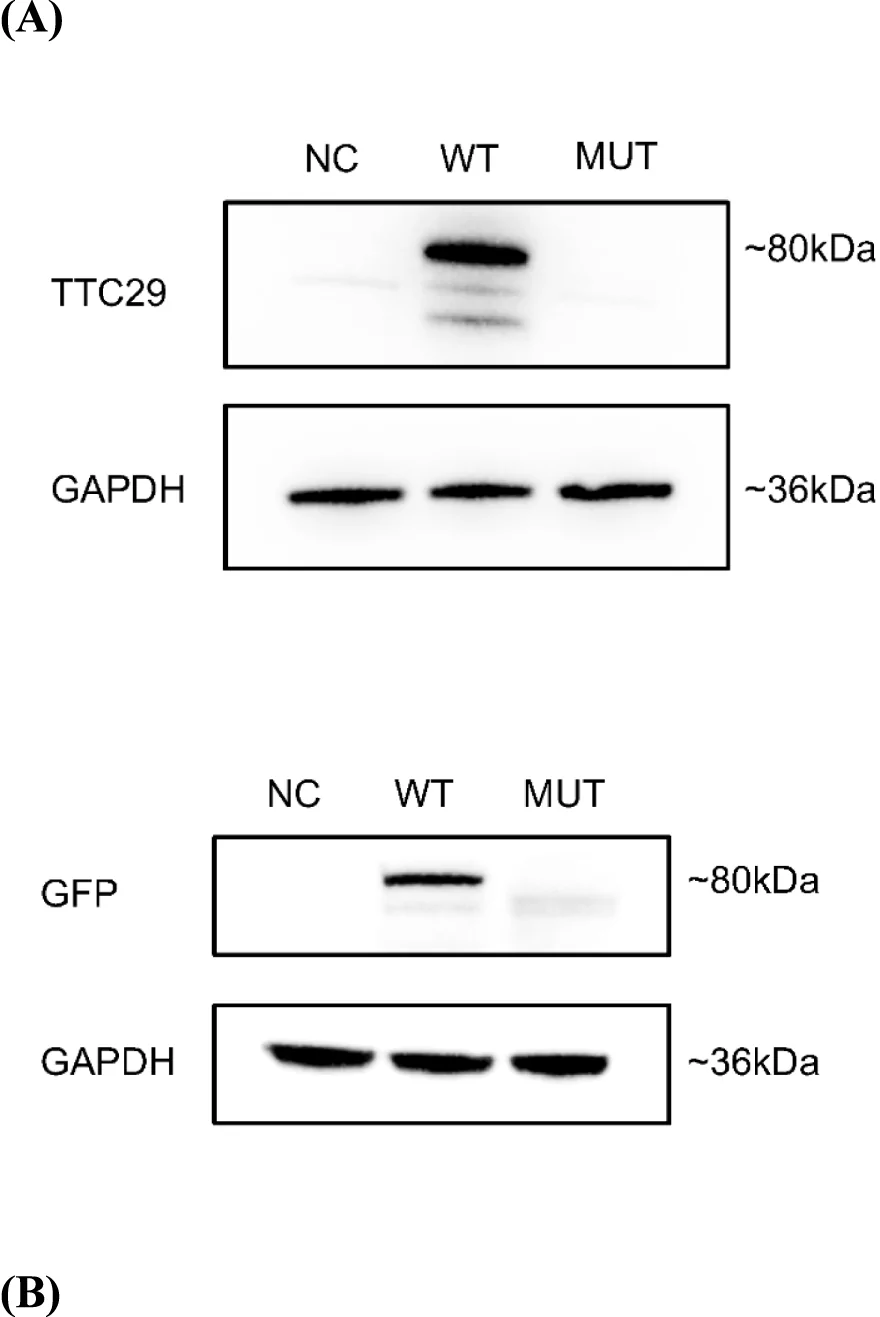
Western blot analysis of TTC29-EGFP expression. Western blotting with anti-TTC29 **(A)** and anti-GFP **(B)** antibodies showed the expected full-length TTC29-EGFP fusion protein in the WT group, whereas the corresponding band was markedly reduced or undetectable in the MUT group. GAPDH served as the loading control. NC, empty vector control; WT, wild-type; MUT, mutant. Full uncropped blot images with molecular-weight markers are provided in Supplementary Figure S1.

To evaluate the effect of c.121dupA on TTC29 protein expression, wild-type and mutant TTC29-EGFP constructs were generated and transfected into HEK293 T cells, with empty vector as a negative control. At 48 h after transfection, GFP fluorescence was readily observed in cells expressing wild-type TTC29, whereas cells expressing mutant TTC29 showed markedly weaker fluorescence (Figure 5). Western blotting further supported this observation. When probed with the TTC29 antibody, an approximately 80-kDa band was detected in the wild-type group, whereas the corresponding band was markedly reduced or undetectable in the mutant group. A similar pattern was observed using the GFP antibody: the approximately 80-kDa TTC29-EGFP fusion protein was detected in the wild-type group but not in the mutant group. GAPDH expression was comparable among groups (Figure 6). Because c.121dupA introduces a premature termination codon upstream of the C-terminal EGFP tag, the reduced signal in the mutant group was consistent with impaired production of the expected TTC29 expression product. The precise molecular basis of this observation remains to be determined.

### 3.6 ICSI outcomes

The female partner was 34 years old, with a body mass index of 22.03 kg/m^2^, an anti-Müllerian hormone level of 3.71 ng/mL, a basal follicle-stimulating hormone level of 8.99 IU/L, and an antral follicle count of 12.

In the first cycle, a follicular-phase long-acting GnRH agonist protocol was used. Six oocytes were retrieved, including five metaphase II (MII) oocytes and one metaphase I oocyte. Among the five MII oocytes, three showed normal 2PN fertilization, corresponding to a normal fertilization rate of 60% (3/5); one showed 1PN fertilization, and one degenerated. All three normally fertilized embryos underwent cleavage. On day 3, two embryos had developed to the 7-cell stage with grade 2 morphology and 10% fragmentation and were transferred. The third normally fertilized embryo had developed to the 7-cell stage with 30% fragmentation and was not used. The 1PN embryo developed to the 5-cell stage with 35% fragmentation and was also not used. In the second cycle, a GnRH antagonist protocol was used. Two MII oocytes were retrieved, and both showed normal 2PN fertilization and subsequent cleavage. On day 3, one embryo had reached the 8-cell stage with grade 2 morphology and 10% fragmentation and was transferred, whereas the other had reached the 4-cell stage with 30% fragmentation and was not used.

Thus, all five normally fertilized embryos obtained across the two cycles underwent cleavage, and no consistent stage-specific developmental arrest was observed. However, some embryos showed slower cleavage and/or increased fragmentation. A total of three day-3 embryos were transferred, but no clinical pregnancy was achieved (Table 2). Given the limited number of oocytes and transferred embryos, this outcome should be interpreted cautiously and cannot be attributed specifically to the identified TTC29 variant.

**TABLE 2:** ICSI outcomes of the couple in two treatment cycles.

Subjects	Cycle 1	Cycle 2
Male age (years)	36	36
Female age (years)	34	34
Ovarian stimulation protocol	Follicular-phase long-acting GnRH agonist protocol	GnRH antagonist protocol
No. of oocytes retrieved	6	2
No. of MII oocytes	5	2
No. of normally fertilized oocytes (2PN)	3	2
Normal fertilization rate (%)	60.0 (3/5)	100.0 (2/2)
Cleavage rate of 2PN embryos (%)	100.0 (3/3)	100.0 (2/2)
No. of available day-3 embryos	2	1
Available embryo rate (%)	66.7 (2/3)	50.0 (1/2)
No. of embryos transferred	2	1
Morphology of transferred embryos	Two 7-cell, grade 2 embryos with 10% fragmentation	One 8-cell, grade 2 embryo with 10% fragmentation
Implantation rate (%)	0	0
Clinical pregnancy rate (%)	0	0
Miscarriage rate (%)	-	-

In cycle 1, one additional 1PN, embryo developed to the 5-cell stage with 35% fragmentation and was not used. One normally fertilized 2PN, embryo developed to the 7-cell stage with 30% fragmentation and was not used. In cycle 2, one normally fertilized embryo developed to the 4-cell stage with 30% fragmentation and was not used. Normal fertilization and available embryo rates were calculated using MII, oocytes and normally fertilized 2PN, embryos as the respective denominators; MII, metaphase II; 2PN, two pronuclei; GnRH, gonadotropin-releasing hormone.

## 4 Discussion

In this study, we identified the novel *TTC29* frameshift variant c.121dupA (p.Ile41Asnfs*21) in an apparently homozygous state in an infertile man with total asthenozoospermia. The patient showed complete loss of progressive and non-progressive sperm motility, with 100% immotile spermatozoa. Sanger sequencing showed an apparently homozygous sequence pattern in the proband and a heterozygous state in his father, findings compatible with an autosomal recessive mode of inheritance. Retrospective exon-level copy-number analysis of the original WES data showed no evidence of a deletion involving *TTC29*, arguing against pseudohomozygosity caused by a large deletion on the second allele. Nevertheless, because maternal DNA and residual biological material for independent dosage testing were unavailable, true homozygosity and the maternal origin of the second allele could not be definitively confirmed. Representative sperm immunofluorescence images showed a visibly weaker TTC29 signal in the proband, whereas heterologous expression analysis showed little or no detectable production of the expected TTC29 expression product from the mutant construct. Together, these findings are consistent with a deleterious effect of c.121dupA on TTC29 protein production or localization, although the sperm immunofluorescence observation was qualitative.

Biallelic loss-of-function variants in *TTC29* have been associated with severe asthenozoospermia, abnormal sperm flagellar morphology, and male infertility, while *Ttc29*-deficient mice exhibit impaired sperm motility and reduced fertility (Liu C. et al., 2019; Lorès et al., 2019). TTC29 is an evolutionarily conserved axonemal protein required for normal sperm flagellar function, and TTC29 deficiency has been associated with reduced signals of the IFT-B-related proteins TTC30 A and IFT52, supporting a potential role in intraflagellar transport and axonemal assembly (Liu C. et al., 2019; Lorès et al., 2019). In addition to flagellar abnormalities, sperm-head and acrosomal defects have been described in individuals carrying biallelic *TTC29* variants, suggesting that the phenotypic consequences of *TTC29* disruption may extend beyond the flagellum (Dai et al., 2022). Furthermore, the reported interaction of TTC29 with ZMYND12 and DNAH1 supports its involvement in a broader axoneme-associated protein network (Dacheux et al., 2023).

Previous genetic studies have identified several biallelic *TTC29* variants in men with severe asthenozoospermia or MMAF, including nonsense, frameshift, and canonical splice-site variants (Liu C. et al., 2019; Lorès et al., 2019; Dai et al., 2022) (Table 3). Severe impairment or complete loss of progressive sperm motility together with multiple flagellar abnormalities represents a common phenotypic feature, although the severity and spectrum of morphological defects vary among affected individuals (Liu C. et al., 2019; Lorès et al., 2019; Dai et al., 2022). Additional sperm-head and acrosomal abnormalities have also been described in an individual carrying compound heterozygous *TTC29* variants (Dai et al., 2022). The c.121dupA variant identified in the present study is located near the N-terminus of TTC29 and upstream of the predicted TPR domains. The associated total asthenozoospermia and extensive axonemal and peri-axonemal abnormalities are generally consistent with the phenotypic spectrum of previously reported *TTC29*-related infertility. Although the reported *TTC29* variants are distributed across different regions of the coding sequence, severe asthenozoospermia and MMAF have been observed with both N-terminal truncating variants and variants located farther downstream (Liu C. et al., 2019; Lorès et al., 2019; Dai et al., 2022). The c.121dupA variant identified here lies near the N-terminus, upstream of all predicted TPR domains, and is associated with total asthenozoospermia and extensive flagellar ultrastructural abnormalities. Thus, the currently available cases do not indicate a simple relationship between variant position and phenotypic severity, although additional cases will be required to define genotype–phenotype correlations more precisely.

**TABLE 3:** Reported human *TTC29* variants, phenotypes, and ICSI outcomes.

Study	Variant (location)	Type/zygosity	Phenotype	ICSI outcome
Lorès et al., 2019 (n = 3)	c.176 + 1G>A; p.Tyr60* (splice donor)	SS/Hom	MMAF; severe AS	Not reported
Lorès et al., 2019 (n = 1)	c.330_334delGGAGG; p.Glu111Alafs* (N-terminal)	FS/Hom	MMAF; PR 0%	Not reported
Lorès et al., 2019 (n = 1)	c.750C>A; p.Tyr250*	NS/Hom	MMAF; PR 0%	Not reported
Liu C. et al., 2019 (n = 1)	c.1107C>G; p.Tyr369*	NS/Hom	MMAF; PR 0%	Clinical pregnancy achieved
Liu C. et al., 2019 (n = 1)	c.412_425del; p.Asp138Leufs*10 (N-terminal)	FS/Hom	MMAF; PR 0%	No clinical pregnancy
Liu C. et al., 2019 (n = 1)	c.1107C>G; p.Tyr369*/c.977 + 1G>T; p.Ser326Profs*8	NS + SS/CH	MMAF; PR 0%	Clinical pregnancy achieved
Dai et al., 2022 (n = 1)	c.1185C>G; p.Tyr395*/c.254 + 1G>A	NS + SS/CH	MMAF + head/acrosome defects	No clinical pregnancy after two cycles

Variant nomenclature and transcript accession numbers are presented as originally reported in the corresponding studies. AS, asthenozoospermia; CH, compound heterozygous; FS, frameshift; Hom, homozygous; MMAF, multiple morphological abnormalities of the sperm flagella; NS, nonsense; PR, progressive motility; SS, splice-site.

At the molecular level, c.121dupA is predicted to introduce a premature termination codon near the N-terminus, upstream of the predicted TPR domains. Frameshift variants of this type may lead to transcript degradation through nonsense-mediated mRNA decay or to the production of truncated proteins (Chang et al., 2007). In the present heterologous expression system, the expected approximately 80-kDa TTC29-EGFP product was markedly reduced or undetectable in the mutant group. As the constructs were generated from intron-free cDNA, this system does not fully recapitulate endogenous transcript processing. The observed reduction may therefore reflect the combined effects of altered transcript abundance, premature translational termination, and/or reduced stability of a truncated product. TTC29 has also been implicated in an axoneme-associated protein network involving ZMYND12 and DNAH1 (Dacheux et al., 2023). Because c.121dupA introduces a premature termination codon upstream of all predicted TPR domains, which are commonly involved in protein–protein interactions, the variant may impair TTC29-mediated interactions within this network or affect the stability of associated axonemal complexes. Such disruption could contribute to the extensive axonemal disorganization and severe loss of sperm motility observed in the proband. However, protein–protein interactions were not directly assessed in the present study, and further studies will be required to clarify both the molecular basis of the reduced expression and the potential effect of c.121dupA on the ZMYND12–TTC29–DNAH1-associated network.

At the ultrastructural level, MMAF is characterized by heterogeneous defects involving both the axonemal core and peri-axonemal structures, including disruption of the canonical “9 + 2” organization and abnormalities of the outer dense fibers, mitochondrial sheath, or fibrous sheath (Ben Khelifa et al., 2014; Nsota Mbango et al., 2019; Zhou et al., 2024). Variants in genes such as *DNAH1*, *CFAP43*, *CFAP44*, *CFAP69*, *CFAP65*, *WDR66*, *AK7*, and *TTC21A* can result in similar sperm flagellar phenotypes, underscoring the genetic heterogeneity of severe sperm motility disorders (Ben Khelifa et al., 2014; Amiri-Yekta et al., 2016; Tang et al., 2017; Dong et al., 2018; Kherraf et al., 2018; Liu W. et al., 2019; Li et al., 2020; Chang et al., 2024). In the present study, TEM revealed severe abnormalities in both the midpiece and principal piece of the proband’s sperm flagella. Quantitative analysis further showed that 96.1% of the proband’s midpiece cross-sections and 97.1% of the principal-piece cross-sections were abnormal, whereas the corresponding proportions in the fertile control were 5.3% and 4.0%, respectively. Isolated CP abnormalities were the most frequent single-defect category, and a substantial proportion of sections showed complex axonemal and peri-axonemal disorganization. Midpiece cross-sections showed an irregular mitochondrial sheath, eccentric axonemes, and disorganized peripheral microtubule doublets and outer dense fibers, whereas principal-piece cross-sections exhibited asymmetric and disorganized fibrous sheaths together with marked disruption of axonemal organization. The central microtubule pair was absent or poorly defined in representative sections, and the canonical “9 + 2” architecture was not preserved. These findings are consistent with previously reported *TTC29*-associated ultrastructural defects, including disorganization of peripheral microtubules, misarrangement of outer dense fibers, and loss of the central pair (Liu C. et al., 2019; Lorès et al., 2019; Dai et al., 2022). Together with the visibly weaker TTC29 signal observed in representative spermatozoa and the heterologous expression findings, these quantitative ultrastructural data are consistent with an adverse effect of c.121dupA on sperm flagellar assembly or stability.

The proband did not show obvious symptoms suggestive of primary ciliary dyskinesia, such as chronic sinusitis, recurrent respiratory infection, or situs inversus. This is consistent with many cases of MMAF in which the phenotype is largely confined to the male reproductive system (Nsota Mbango et al., 2019; Sironen et al., 2020). However, because some cilia-related genes can affect both motile cilia and sperm flagella, clinical assessment of men with severe sperm motility defects should still include careful review of respiratory and other ciliopathy-related symptoms.

The reproductive outcome in this case also provides useful clinical information regarding ICSI in TTC29-associated infertility. In the first cycle, three of five MII oocytes showed normal fertilization, and all three 2  P N embryos underwent cleavage. Two 7-cell day-3 embryos with grade 2 morphology and 10% fragmentation were transferred, whereas the remaining normally fertilized embryo showed 30% fragmentation. In the second cycle, both MII oocytes showed normal fertilization and cleavage, yielding one 8-cell embryo with grade 2 morphology and 10% fragmentation and one 4-cell embryo with 30% fragmentation. Thus, no consistent stage-specific developmental arrest was observed, although some embryos showed slower cleavage and/or increased fragmentation.

Previous reports on ICSI outcomes in TTC29-associated infertility are not uniform. In three men carrying biallelic TTC29 variants, the median fertilization, cleavage, eight-cell formation, and blastocyst formation rates were 74.1%, 100.0%, 40.9%, and 31.8%, respectively, and clinical pregnancies were achieved in two couples. These outcomes did not differ significantly from those observed in DNAH1-associated MMAF or oligoasthenoteratozoospermia controls (Liu C. et al., 2019). In another patient carrying compound heterozygous TTC29 variants, fertilization and day-3 embryo development were achieved after ICSI, but no clinical pregnancy was obtained after two treatment cycles, and possible female-related factors could not be excluded (Dai et al., 2022). At the broader MMAF level, comparable fertilization and clinical pregnancy outcomes have been reported between MMAF and non-MMAF groups (Ferreux et al., 2021), whereas a more recent cohort showed reduced embryo developmental potential and lower cumulative pregnancy rates in men with MMAF (Long et al., 2024). These findings suggest that reproductive outcomes following ICSI in MMAF may vary among patients and genetic backgrounds. In the present case, the female partner was 34 years old, and her AMH, basal FSH, and AFC did not suggest an obvious reduction in ovarian reserve. Nevertheless, only eight oocytes and three transferable day-3 embryos were obtained across the two cycles. The absence of clinical pregnancy may therefore reflect the combined influence of embryo number and quality, oocyte competence, endometrial receptivity, transfer-related factors, and other couple-specific factors. Accordingly, the absence of clinical pregnancy cannot be attributed specifically to c.121dupA or used to infer an unfavorable prognosis for *TTC29*-associated infertility.

This study has several limitations. First, the study was based on a single family, and complete segregation analysis could not be performed because the proband’s mother had died and no maternal biological sample was available. The proband was an only child, and no informative maternal relatives were available for additional genetic testing. Although retrospective exon-level read-depth-based analysis of the original WES data showed no evidence of a deletion involving *TTC29*, no residual blood, genomic DNA, or other biological material was available for independent dosage testing or breakpoint analysis. Therefore, a small or technically undetectable deletion involving the second allele cannot be completely excluded, and the maternal origin of the second c.121dupA allele could not be directly confirmed. Identification and characterization of additional unrelated patients carrying *TTC29* variants, particularly c.121dupA, will be important to further strengthen variant-specific genetic evidence and define the associated phenotypic spectrum. Second, the sperm immunofluorescence findings were based on representative images without quantitative fluorescence-intensity analysis, which limits the assessment of the magnitude and variability of the reduction in TTC29 signal. In addition, the heterologous HEK293 T expression system, based on intron-free cDNA constructs, does not fully recapitulate endogenous TTC29 transcript processing or sperm-specific flagellar assembly. Further studies using appropriate germ-cell systems and variant-specific *in vivo* models may help clarify the effects of c.121dupA on TTC29 expression, spermiogenesis, protein interactions, and sperm flagellar assembly and motility.

In summary, we identified a novel *TTC29* frameshift variant, c.121dupA (p.Ile41Asnfs*21), in a man with total asthenozoospermia and primary infertility. The variant was associated with extensive ultrastructural abnormalities of the sperm flagellar midpiece and principal piece, a visibly weaker TTC29 signal in representative spermatozoa from the proband, and impaired production of the expected TTC29 expression product in a heterologous cell system. These findings expand the mutational and ultrastructural phenotype spectrum of *TTC29*-related male infertility and support genetic testing of *TTC29* and other flagellar genes in men with total asthenozoospermia or MMAF-related infertility.

## Data Availability

The datasets presented in this study can be found in online repositories. The names of the repository/repositories and accession number(s) can be found in the article/Supplementary Material.
