# Supplementary material for: A novel frameshift variant in TTC29 associated with total asthenozoospermia and male infertility
Source: Front Cell Dev Biol. 2026 Aug 27;14:1937470. doi: 10.3389/fcell.2026.1937470 (PMC13583038; doi:10.3389/fcell.2026.1937470)
Supplement: Supplementary file 1 [file DataSheet1.pdf]

Supplementary Material

Supplementary Figures

(A)

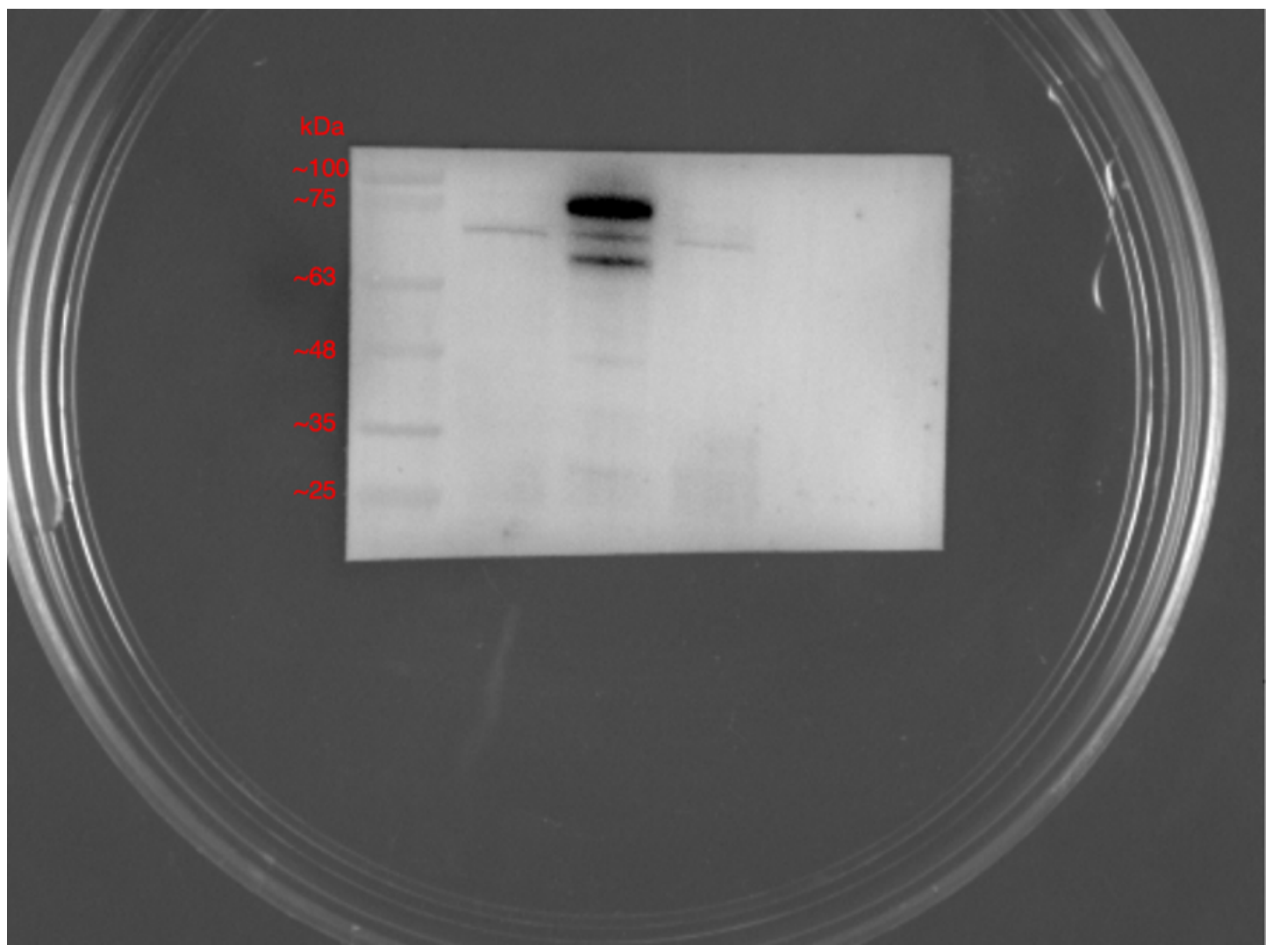

(B)

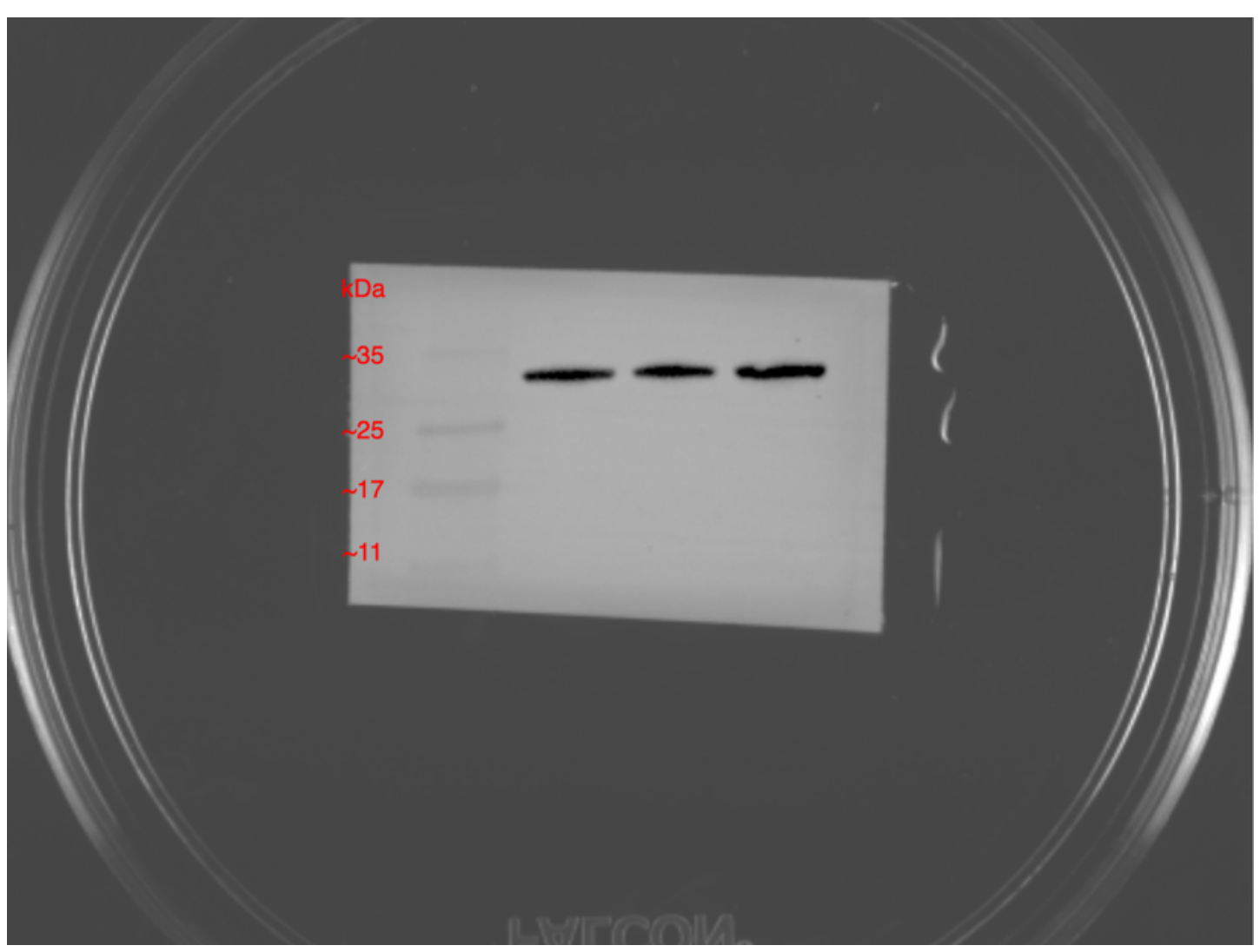

(C)

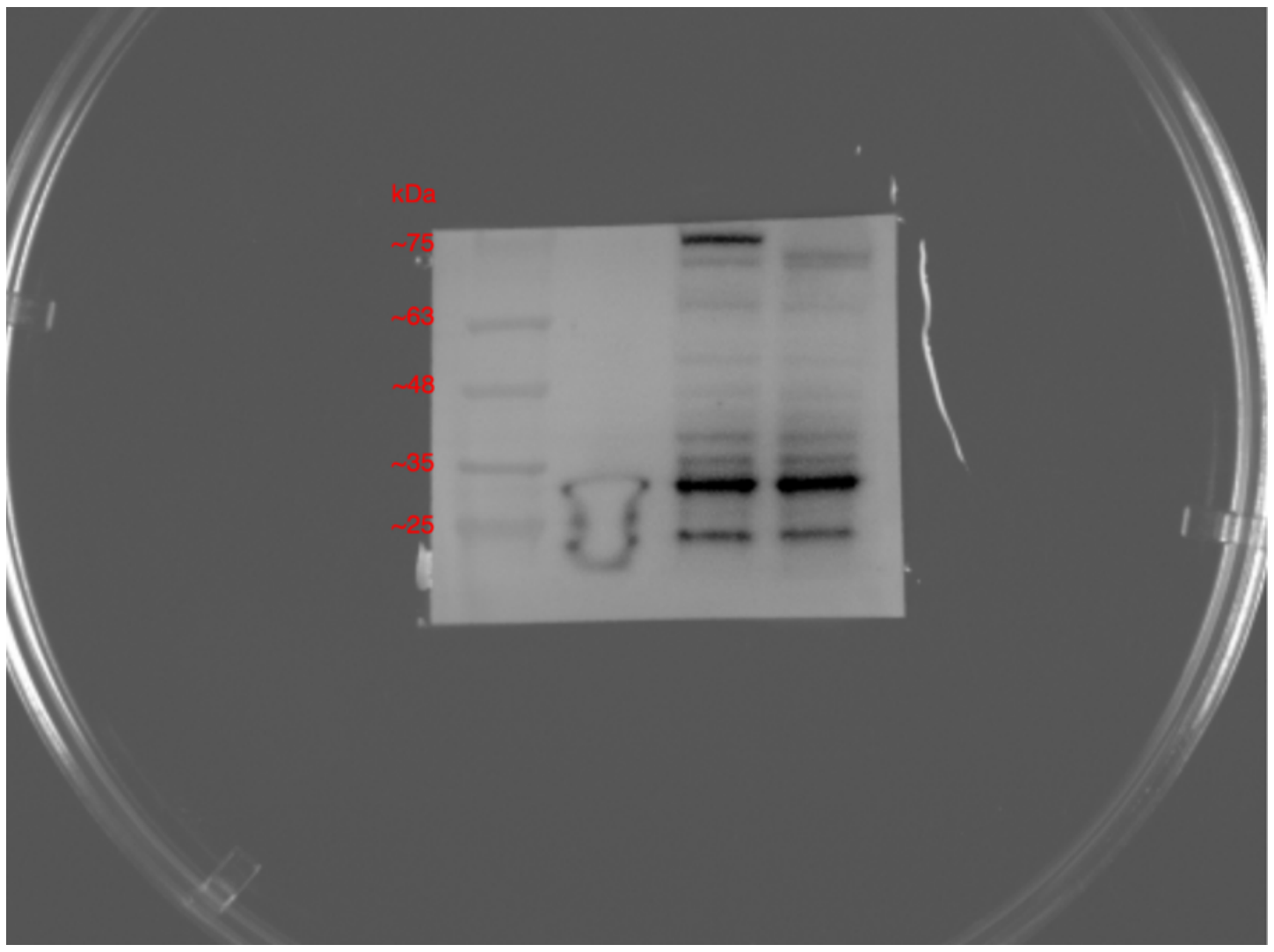

(D)

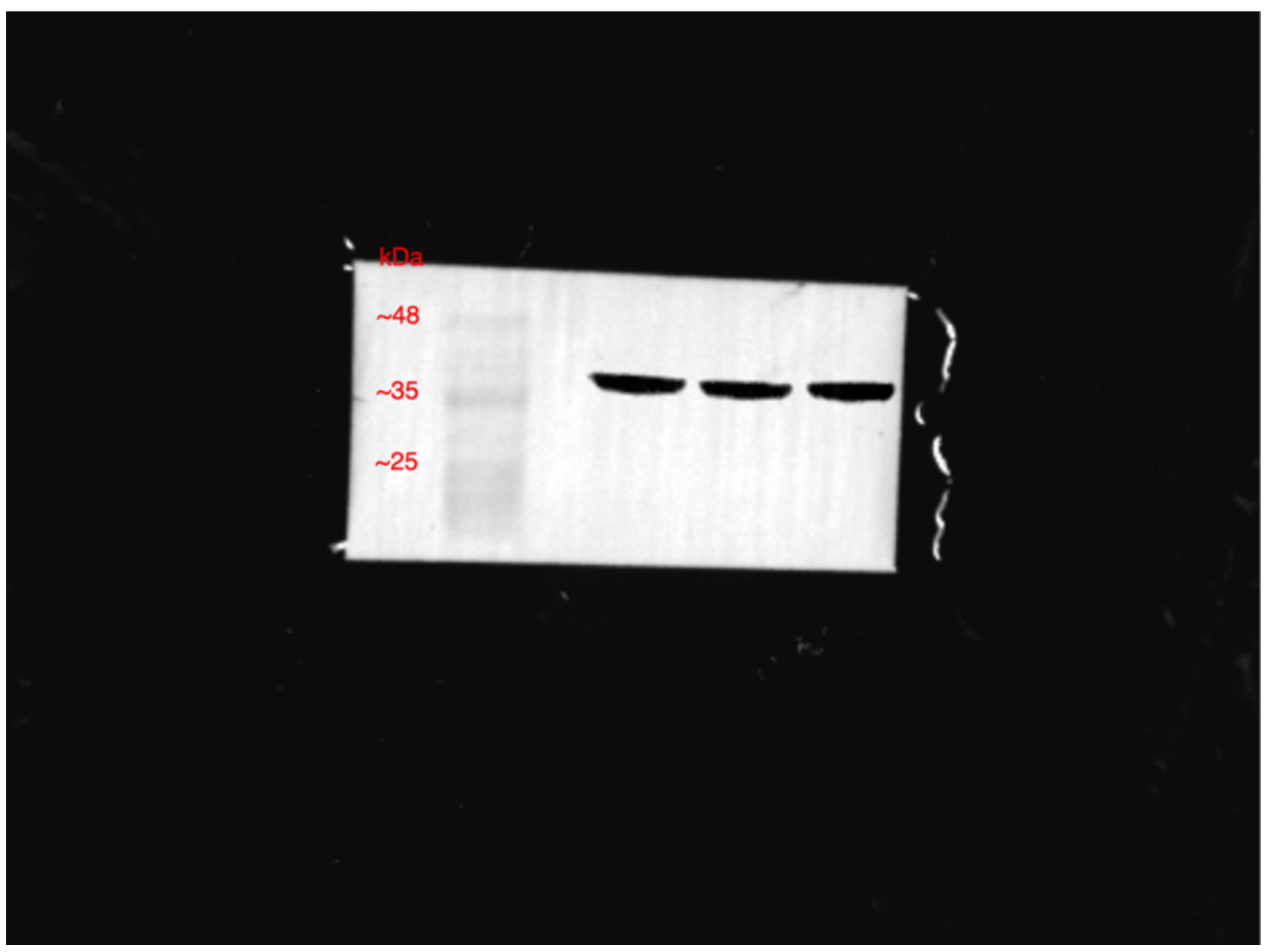

Supplementary Figure 1. Full uncropped Western blot images corresponding to Figure 6. (A) Uncropped Western blot obtained using the anti-TTC29 antibody. (B) Corresponding GAPDH blot used as the loading control. (C) Uncropped Western blot obtained using the anti-GFP antibody. (D) Corresponding GAPDH blot used as the loading control. For each blot, the lanes are arranged from left to right as molecular-weight marker (M), empty-vector control (NC), wild-type TTC29-EGFP (WT), and mutant TTC29-EGFP (MUT). Molecular-weight markers are indicated alongside the blots.
